# Rim lesions are demonstrated in early relapsing–remitting multiple sclerosis using 3 T-based susceptibility-weighted imaging in a multi-institutional setting

**DOI:** 10.1007/s00234-021-02768-x

**Published:** 2021-10-19

**Authors:** Koy Chong Ng Kee Kwong, Daisy Mollison, Rozanna Meijboom, Elizabeth N. York, Agniete Kampaite, Sarah-Jane Martin, David P. J. Hunt, Michael J. Thrippleton, Siddharthan Chandran, Adam D. Waldman, FutureMS consortium

**Affiliations:** 1 Centre for Clinical Brain Sciences, University of Edinburgh, Edinburgh bioQuarter, Chancellor’s Building, 49 Little France Crescent, Edinburgh, EH16 4SB UK; 2 Department of Neurosciences, University of Glasgow, Glasgow, UK

**Keywords:** Multiple sclerosis, Magnetic resonance imaging, Susceptibility-weighted imaging, Rim lesions

## Abstract

**Purpose:**

Rim lesions, characterised by a paramagnetic rim on susceptibility-based MRI, have been suggested to reflect chronic inflammatory demyelination in multiple sclerosis (MS) patients. Here, we assess, through susceptibility-weighted imaging (SWI), the prevalence, longitudinal volume evolution and clinical associations of rim lesions in subjects with early relapsing–remitting MS (RRMS).

**Methods:**

Subjects (*n* = 44) with recently diagnosed RRMS underwent 3 T MRI at baseline (M0) and 1 year (M12) as part of a multi-centre study. SWI was acquired at M12 using a 3D segmented gradient-echo echo-planar imaging sequence. Rim lesions identified on SWI were manually segmented on FLAIR images at both time points for volumetric analysis.

**Results:**

Twelve subjects (27%) had at least one rim lesion at M12. A linear mixed-effects model, with ‘subject’ as a random factor, revealed mixed evidence for the difference in longitudinal volume change between rim lesions and non-rim lesions (*p* = 0.0350 and *p* = 0.0556 for subjects with and without rim lesions, respectively). All 25 rim lesions identified showed T1-weighted hypointense signal. Subjects with and without rim lesions did not differ significantly with respect to age, disease duration or clinical measures of disability (*p* > 0.05).

**Conclusion:**

We demonstrate that rim lesions are detectable in early-stage RRMS on 3 T MRI across multiple centres, although their relationship to lesion enlargement is equivocal in this small cohort. Identification of SWI rims was subjective. Agreed criteria for defining rim lesions and their further validation as a biomarker of chronic inflammation are required for translation of SWI into routine MS clinical practice.

**Supplementary Information:**

The online version contains supplementary material available at 10.1007/s00234-021-02768-x.

## Introduction

Multiple sclerosis (MS) is a chronic neurological condition affecting over 2 million individuals worldwide [1]. MS is an immune-mediated disease characterised by inflammation, demyelination, neurodegeneration and remyelination. Most patients initially diagnosed with relapsing–remitting MS (RRMS), the most common form of MS, experience two distinct clinical phases, reflecting a dominant role for distinct pathological processes. Inflammation drives activity during the relapsing–remitting stage, with neurodegeneration being predominant in progressive MS characterised by accumulating disability [2].

Whilst the reference standard for pathological characterisation is histological analysis of biopsy or, more commonly, autopsy material [3–5], recent progress in imaging raises the possibility of non-invasive and longitudinal measurement and ‘pathological’ characterisation of lesions. For instance, susceptibility-based magnetic resonance imaging (MRI) provides support for the paramagnetic properties of certain MS lesions [6–8]. These lesions may be identified on susceptibility-weighted imaging (SWI) by a hyperintense core surrounded by a partial or complete rim of hypointense signal. The high tissue susceptibility observed at the edge of rim lesions has been histologically shown to correspond to iron deposition and a high density of activated myeloid cells, suggesting the involvement of iron-laden macrophages/microglia [9–11]. The origin of iron in myeloid cells is still unclear, although damage to myelin and oligodendrocytes may result in the release of iron into the interstitium and subsequent engulfment by macrophages and microglia [12].

Rim lesions have been found to coincide with regions of hypointensity on T1-weighted spin-echo sequences, also known as ‘T1 black holes’, as well as with hypointensity on fluid-attenuated inversion recovery (FLAIR), both of which are associated with substantial tissue damage [13, 14]. Rim lesions appear to experience significant growth over time compared with lesions without rims, supporting histological evidence that they could reflect a state of chronic inflammatory demyelination due to macrophage/microglial activity [10, 11]. Iron-laden rims are also rarely found around remyelinated plaques [10]. Against this background of combined pathological and radiological evidence, rim lesions have been argued to represent a potential marker of chronic or low-level inflammation in MS. Rim lesions are also not present in ‘mimic’ conditions such as neuromyelitis optica spectrum disorder (NMOSD), Susac syndrome and cerebrovascular disease [15–20].

The clinical correlates of rim lesions are unclear, as they are observed in clinically isolated syndrome (CIS), radiologically isolated syndrome (RIS) as well as RRMS and progressive disease [21–23]. However, recent results suggest that non-gadolinium enhancing rim lesions, also known as ‘chronic active lesions’, are linked to a more severe disease phenotype [11]. The majority of rim lesion studies in MS have been single-centre and focussed on later stage disease [23]. In this multi-centre study, we sought to evaluate the prevalence of rim lesions and their relationship with ‘T1-w hypointense lesions’ in recently diagnosed RRMS patients using SWI at 3 T.

## Methods

### Subjects

Subjects (*n* = 44) were recruited from a multi-centre prospective observational cohort study for people recently diagnosed with RRMS. The inclusion criteria were as follows: diagnosis of RRMS according to 2010 McDonald criteria [24], within 6 months of diagnosis at baseline assessment (M0), age ≥ 18 years at baseline assessment, disease-modifying therapy (DMT) not prescribed prior to baseline assessment, no contraindication to MR brain imaging. Participants attend two clinic visits, approximately 12 months apart for clinical examinations, brain MR imaging and laboratory tests. SWI was carried out at the 1-year time point (M12).

### Clinical assessment

Motor and cognitive disability of participants are assessed via various measures including the Expanded Disability Status Scale (EDSS), the Timed 25-Foot Walk Test (T25FWT), the Paced Auditory Serial Addition Test (PASAT), the Symbol Digit Modalities Test (SDMT) and the Multiple Sclerosis Functional Composite (MSFC) score. Plasma neurofilament (NfL) levels are obtained from participants at M0 using a single-molecule ELISA. The assay is run on a 4-Plex "A" Kits Quanterix and Simoa SR-XTM benchtop instrument.

### MRI acquisition

MRI was performed at three research sites on Prisma 3 T MRI systems (Siemens Healthcare, Erlangen, De) using either a 32-channel or 20-channel receive head coil and with a maximum gradient strength of 80 mT/m. The brain imaging protocol for both timepoints (M0 and M12) includes 3D volume T1-weighted, 2D axial PD/T2-weighted (dual echo), 3D volume FLAIR and 2D axial FLAIR sequences (please see Supplementary Table S1 for detailed acquisition parameters). SWI was acquired at M12; magnitude, phase and susceptibility-weighted images were acquired using a 3D segmented gradient-echo echo-planar imaging sequence (repetition time (TR)/echo time (TE) = 64/35 ms, flip angle = 10 degrees, voxel size = 0.65 mm isotropic, acquisition time = 7:08 m:ss) [25].

### Data analysis

#### Identification of rim lesions

Following conversion of DICOM images to NIfTI format using the dcm2niix tool [26], rigid body registration (6 degrees of freedom) of follow-up 2D FLAIR to SWI images was performed using the FMRIB’s Linear Image Registration Tool (FLIRT) software [27, 28]. Hyperintense white matter lesions (WMLs) were identified on registered 2D FLAIR and assessed in all three anatomical planes on corresponding SWI images using ITK-SNAP Version 3.8.0 [29]. Rim lesions were defined as lesions that were hyperintense on FLAIR and were characterised on SWI by a hyperintense core partially or completely surrounded by a hypointense rim. Possible rim lesions were identified by one trained observer and reviewed by a senior neuroradiologist. SWI images were also independently assessed for rim lesions by a second neuroradiologist, after which the final rim lesion count for each subject was determined by consensus of all three raters. Lesions with a minimum diameter of less than 3 mm were deemed too small to be reliably assessed for the presence of a rim and were excluded. Lesions located near a high density of veins or considerable juxtacortical signal heterogeneity could not be reliably evaluated and were therefore not considered for inclusion.

#### Identification of ‘T1-w hypointense lesions’ and ‘FLAIR-hypointense core lesions’

After rim lesion identification, T1-weighted images were registered to SWI images. SWI rim lesions were assessed on corresponding registered T1-weighted and 2D FLAIR images and classified as ‘T1-w hypointense lesions’ and ‘FLAIR-hypointense core lesions’, respectively. ‘T1-w hypointense lesions’ were defined as lesions that were hyperintense on FLAIR and corresponded to a hypointense region on T1-weighted images. ‘FLAIR-hypointense core lesions’ were defined as lesions hyperintense on FLAIR, but demonstrating regions of hypointensity or isointensity relative to extralesional white matter, typically in the lesion core. Unregistered T1-weighted images at follow-up were also assessed to determine the total ‘T1-w hypointense lesion’ count. Lesions with a minimum diameter of less than 3 mm were excluded, as these could not be reliably assessed. The ‘T1-w hypointense lesion’ count was performed twice on 10 randomly selected subjects in order to calculate the intraclass correlation coefficient (ICC). T2-weighted WML volume was estimated on 3D FLAIR as a percentage of intracranial volume (ICV).

#### Longitudinal volume analysis

Individual rim lesions were manually segmented on unregistered 3D FLAIR images at both time points, and volumes computed using ITK-SNAP Version 3.8.0 [29]. All rim lesions were included in the longitudinal volume analysis. Distinct non-confluent lesions without rims in (a) subjects with rim lesions and (b) subjects without rim lesions were chosen as controls. Where possible, an equal number of rim and non-rim lesions were selected in each subject to allow for within-participant variability. Lesions that were previously excluded as they could not be reliably assessed for the presence of a rim were not eligible to act as control. Manual segmentation was performed twice on 10 randomly selected lesions (either rim or non-rim lesion) in order to determine the ICC.

### Statistical analysis

Study participants were first classified into those with rim lesions and those without rim lesions at follow-up. Differences between the two groups with respect to demographic and clinical characteristics were assessed via statistical tests such as the Mann–Whitney *U* test and Fisher’s exact test according to variable type. Adjustment for multiple comparisons was not performed for demographic and clinical comparisons due to the exploratory nature of the study, although exact uncorrected p-values are reported. The relationship between rim lesion count and both ‘T1-w hypointense lesion’ count and WML volume was assessed by Spearman’s correlation. Differences in lesion volume between baseline and follow-up were evaluated using the Kruskal–Wallis test, with post hoc multiple comparisons using Dunn’s test with Bonferroni adjustment. A linear mixed-effects model was used to assess differences in longitudinal volume change amongst lesion groups, with ‘lesion type’ as a fixed effect and ‘subject’ as a random effect. ICC estimates were calculated based on a single rating, absolute agreement, and two-way mixed-effects model. All statistical analyses were performed using R Version 3.6.3.

## Results

### Demographic and clinical characteristics

Forty-four subjects were included, all of whom were diagnosed with RRMS (Table 1). Seventy-five percent of the cohort was female. Mean age at the time of SWI was 42.6 years (SD = 12.8), while mean times from diagnosis and onset of symptoms to SWI were 494 days (SD = 67) and 1781 days (SD = 1840), respectively. Mean duration of follow-up was 415 days (SD = 50). Median follow-up and baseline EDSS scores were 3.0 (1.0–6.0) and 2.25 (0–6.0), respectively. Median scores at follow-up for other measures of clinical disability including T25FWT, PASAT, SDMT and MSFC are provided in Table 1. Twenty-eight out of 42 subjects (data not available for two subjects) were on DMT at follow-up, with two subjects being on more than one drug. Mean baseline plasma NfL levels were 8.4 pg/mL (SD = 5.2). Individual subject characteristics are provided in Supplementary Table S2.

**Table 1:** Demographic and clinical characteristics of study cohort

	All subjects	Subjects without rim lesions	Subjects with rim lesions	*p* value
Count, No. (%)	44 (100)	32 (72.7)	12 (27.3)	-
Age (years), mean ± SD (range)	42.6 ± 12.8 (22.0–68.3)	44.3 ± 12.5 (22.0–68.3)	37.9 ± 12.8 (23.2–57.9)	0.169*
Female, No. (%)	33 (75)	27 (84.4)	6 (50)	0.045**
Time from diagnosis to SWI (days), mean ± SD (range)^a^	494 ± 67 (382–648)	503 ± 70 (391–648)	473 ± 54.6 (382–541)	0.356*
Time from onset to SWI (days), mean ± SD (range)^b^	1781 ± 1840 (588–9669)	1977 ± 2154 (588–9669)	1350 ± 732 (705–2676)	0.589*
Duration of follow-up (days) mean ± SD (range)	415 ± 50 (363–532)	419 ± 54 (363–532)	403 ± 35 (367–470)	0.693*
Follow-up EDSS, median (range)	3.0 (1.0–6.0)	3.0 (1.0–6.0)	2.75 (1.0–6.0)	0.632*
Baseline EDSS, median (range)	2.25 (0–6.0)	2.25 (0–6.0)	2.25 (1.0–5.5)	0.850*
Change in EDSS, mean ± SD (range)	0.35 ± 0.94 (− 1.5–4.0)	0.25 ± 0.79 (-1.5–2.0)	0.5 ± 1.25 (− 1.0–4.0)	0.467*
T25FWT, median (range)	4.5 (3.5–12.1)	4.5 (3.5–8.1)	4.6 (3.9–12.1)	0.251*
PASAT, median (range)	47.0 (0–60.0)	45.5 (0–59.0)	51.5 (30.0–60.0)	0.126*
SDMT, median (range)	58.0 (26.0–75.0)	57.5 (26.0–72.0)	60.5 (28.0–75.0)	0.823*
MSFC, median (range)	0.31 (− 1.76–1.33)	0.32 (− 1.76–1.33)	0.35 (-1.62–0.83)	0.969*
DMT, No. (%)^c^	28 (66.7)	19 (61.3)	9 (81.8)	0.283**
Alemtuzumab	2 (4.8)	2 (6.5)	0 (0)	
Azathioprine	2 (4.8)	2 (6.5)	0 (0)	
Dimethyl fumarate	14 (33.3)	7 (22.6)	7 (63.6)	
Glatiramer acetate	5 (11.9)	5 (16.1)	0 (0)	
Interferon beta‑1a	3 (7.1)	3 (9.7)	0 (0)	
Other	4 (9.5)	2 (6.5)	2 (18.2)	
Baseline plasma NfL levels (pg/mL), mean ± SD (range)^d^	8.4 ± 5.2 (2.6–24.1)	8.6 ± 5.5 (2.6–24.1)	8.0 ± 4.2 (4.2–16.6)	0.760*

^a^Two missing values

^b^Twelve missing values

^c^Two missing values; Two subjects on two different drugs

^d^One missing value

^*^Statistical testing performed using Mann–Whitney *U* test

^**^Statistical testing performed using Fisher’s exact test

### Rim lesions

**Fig. 1:**
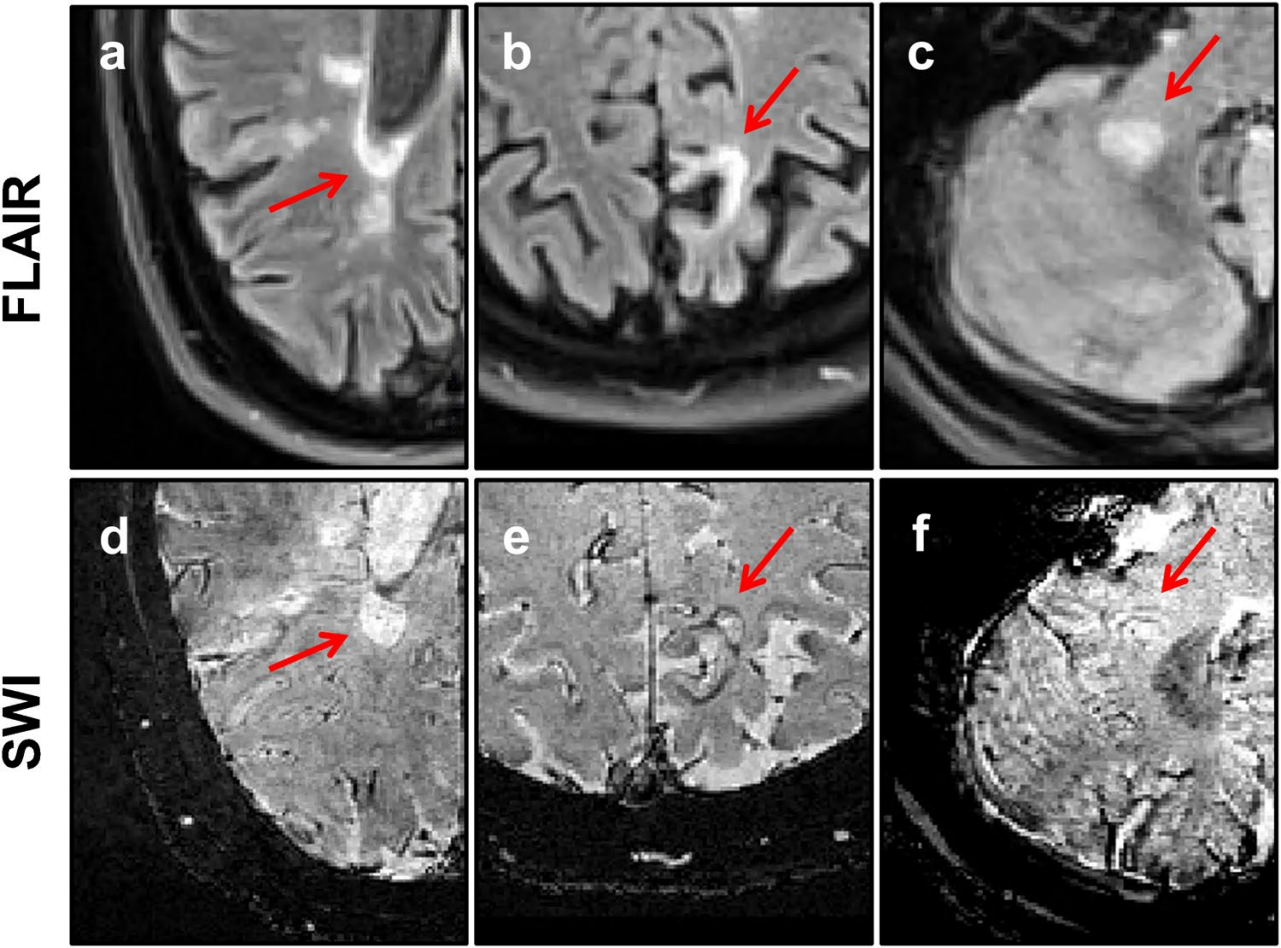
Rim lesions observed at 3 T. SWI and corresponding FLAIR images showing representative examples of rim lesions (indicated by red arrow) in different brain locations including periventricular (**a, d**), juxtacortical (**b, e**) and infratentorial (**c**, **f**) regions

Twelve subjects (27%) demonstrated at least one rim lesion. A total of 25 rim lesions were identified, with the rim lesion count per subject ranging from 1 to 3, except in one subject in which 8 rim lesions were identified (mean [SD] for rim lesion count = 0.6 [1.4]). Rim lesions were predominantly observed supratentorially, with only one infratentorial rim lesion being identified. Examples of SWI rim lesions and corresponding 2D FLAIR images are shown in Fig. 1. Although concordant results were obtained for 9 out of 12 subjects with rim lesions following independent review by the two groups of raters, discussion amongst all three raters was required to reach a consensus regarding the rim lesion count in the remaining 3 subjects.

### ‘T1-w hypointense lesions’ and ‘FLAIR-hypointense core lesions’

**Fig. 2:**
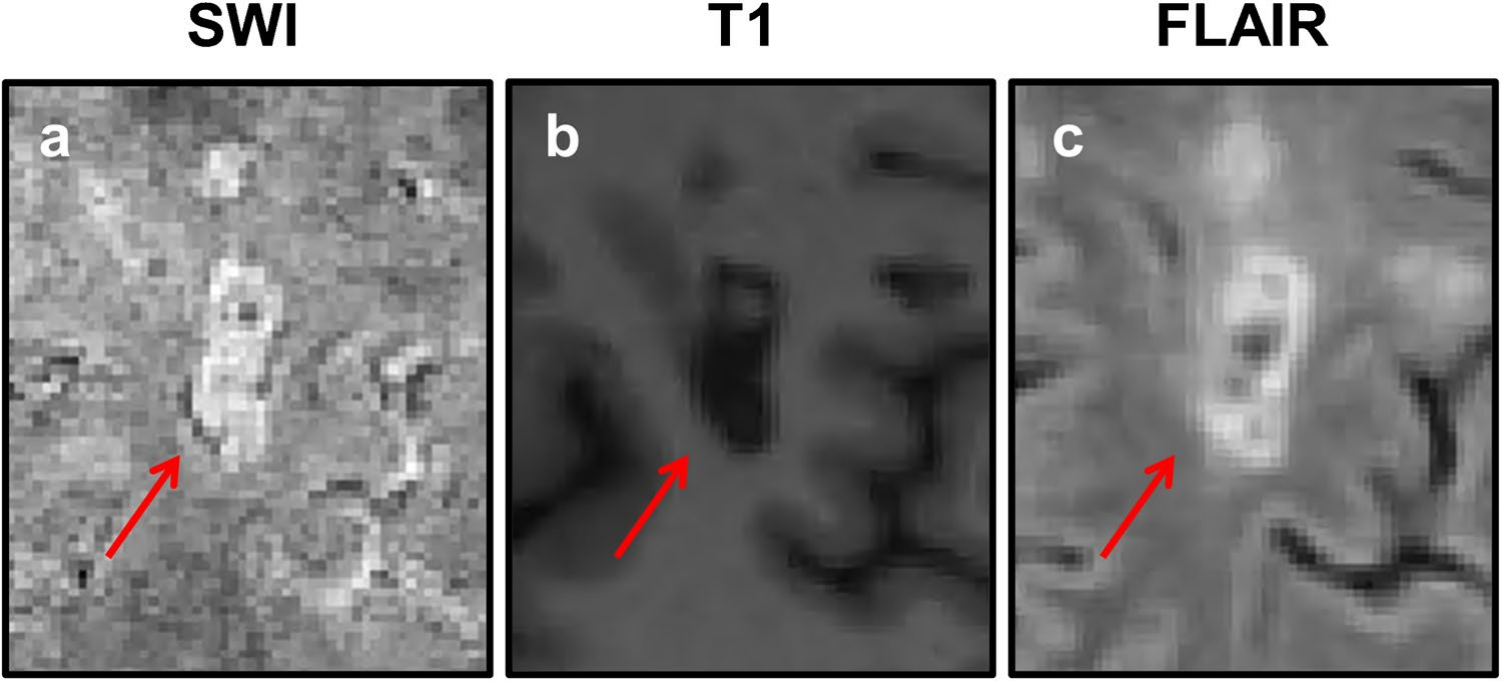
T1 and FLAIR appearance of an SWI rim lesion. All rim lesions observed in our MS cohort, including this white matter lesion (indicated by red arrow) exhibited T1 hypointensity. The lesion shown above is one of ten rim lesions that on FLAIR also appeared hyperintense with a core isointense or in this case, hypointense, relative to extralesional white matter

Assessment of registered T1-weighted images and SWI images revealed that all 25 rim lesions (100%) corresponded to ‘T1-w hypointense lesions’. Ten rim lesions (40%) could further be classified as ‘FLAIR-hypointense core lesions’ on FLAIR images. Figure 2 shows an example of a rim lesion that on T1-weighted image demonstrated hypointensity, and on 2D FLAIR appeared as a hyperintense lesion exhibiting intralesional isointensity. A total of 874 ‘T1-w hypointense lesions’ were observed in all 44 subjects (Mean [SD] for ‘T1-w hypointense lesion’ count = 20 [18]). All subjects had at least one ‘T1-w hypointense lesion’ (Range = 2–116). Rim lesions constituted only a small proportion of total ‘T1-w hypointense lesions’ (2.9%). No significant correlation was observed between rim lesion count and ‘T1-w hypointense lesion’ count (Spearman’s rho = 0.271, *p* value = 0.0752). Estimated ICC with 95% confidence intervals for ‘T1-w hypointense lesion’ count was 0.989 (0.950–0.997). The correlation between rim lesion count and WML volume was non-significant (Spearman’s rho = 0.118, *p* value = 0.4474).

### Assessing group differences

No significant differences were observed at M12 between subjects with rim lesions and subjects without rim lesions with regard to age, disease duration, DMT status, EDSS or any other measures of clinical disability including T25FWT, PASAT, SDMT or MSFC (Table 1). Baseline plasma NfL levels also did not differ between the two groups. Female subjects appeared to constitute a greater proportion of subjects without rim lesions than of subjects with rim lesions (*p* = 0.045).

### Longitudinal volume analysis

Twenty-five rim lesions, 26 non-rim lesions in subjects with rim lesions and 26 non-rim lesions in subjects without rim lesions were selected for longitudinal volume analysis (please see Supplementary Table S3 for more information on lesion selection). Rim lesions were significantly larger than non-rim lesions both in subjects with and without rim lesions both at baseline (mean [SD]/mm^3^ = 489 [253], 152 [108] and 203 [146], respectively; *p* < 0.001) and at follow-up (mean [SD]/mm^3^ = 506 [263], 138 [102] and 165 [103], respectively; *p* < 0.001). Non-rim lesions in subjects with and without rim lesions were not significantly different in size both at baseline and follow-up (*p* = 0.806 and *p* = 0.810, respectively). Estimated ICC with 95% confidence intervals for volume determination by manual segmentation was 0.988 (0.954–0.997).

**Fig. 3:**
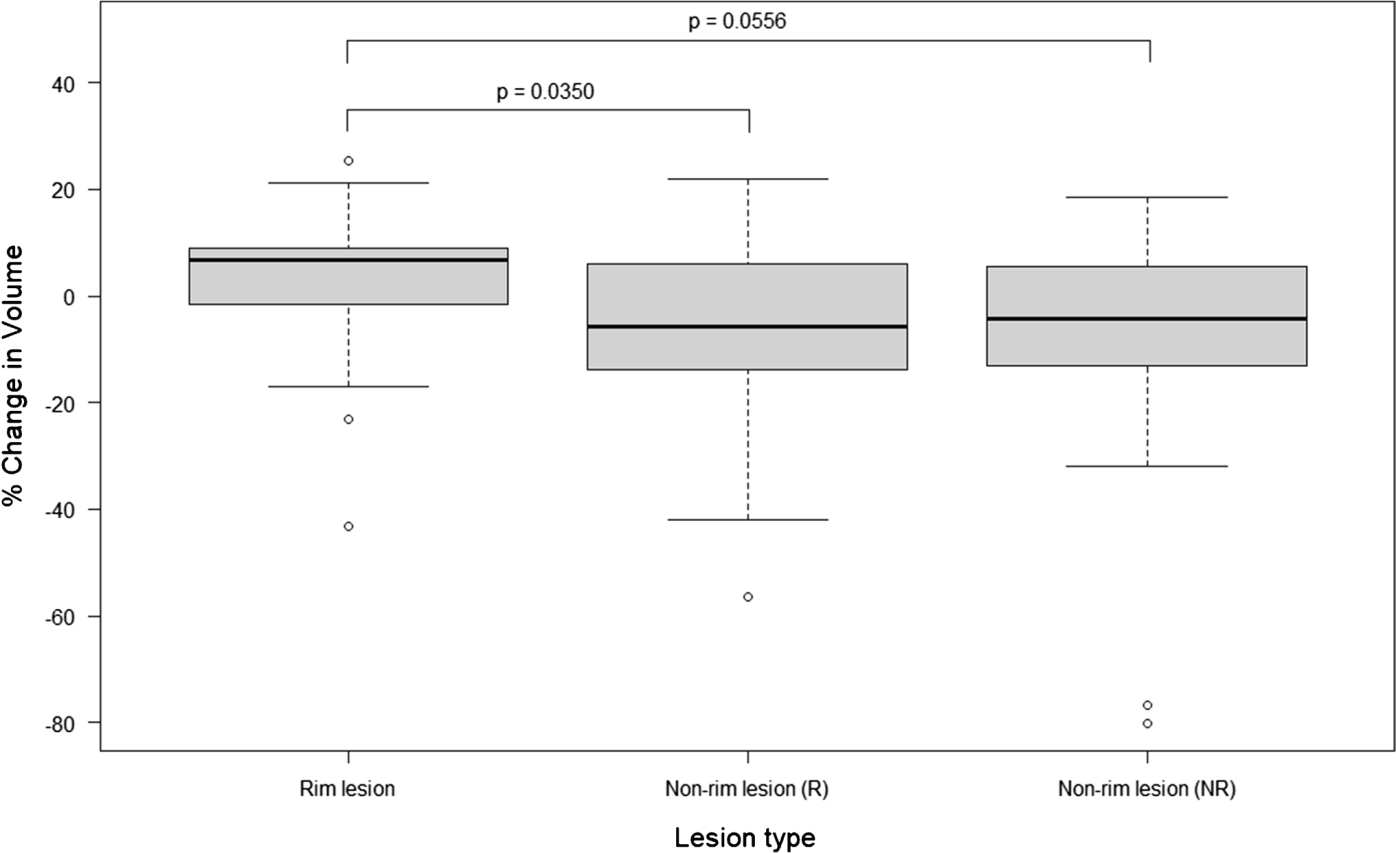
Longitudinal lesion volume change. Boxplots comparing volume changes over 1 year across the three different lesion groups, namely, rim lesions (left), non-rim lesions in subjects with rim lesions (middle) and non-rim lesions in subjects without rim lesions (right)

We found mixed evidence for the difference in 1-year percentage longitudinal volume change between rim lesions and non-rim lesions (*p* = 0.0350 and *p* = 0.0556 for subjects with and without rim lesions, respectively). Percentage changes in lesion volumes are shown in Fig. 3. We also arbitrarily categorised lesions, taking into account the margin of error during manual segmentation, as shrinking (*x* <  − 5%), steady (− 5% < *x* < 5%) or enlarging (*x* > 5%). Although shrinking, steady and enlarging subtypes were observed in all three lesion groups, enlarging lesions constituted a greater proportion of rim lesions (60%) compared to non-rim lesions (27% for both subjects with and without rim lesions). A breakdown of lesion groups into the different subtypes is available from the Supplementary Material.

## Discussion

In this study involving a cohort of recently diagnosed subjects with RRMS, we observed a rim lesion prevalence of 27%, with 12 out of 44 subjects having at least one. This accords with other recently published data [23] and suggests that rim lesions are a feature of early MS, although less common than in patients with longer disease durations. Our study adds to the growing body of literature on SWI at 3 T [11, 22, 23, 30, 31] and furthermore demonstrates the feasibility of multi-centre acquisition, albeit using a single manufacturer MRI platform in this instance. Further studies across different manufacturer platforms will be necessary for validation in large cohort studies towards future clinical implementation.

Notably, all 25 of the rim lesions identified in our study corresponded to ‘T1-w hypointense lesions’. Rim lesions comprised only a very small proportion of the overall number of ‘T1-w hypointense lesions’, however, and no significant correlation was observed between rim lesion count and ‘T1-w hypointense lesion’ count. Pathological correlation studies have shown ‘T1-w hypointense lesions’ characterised on spin-echo sequences as ‘T1 black holes’ to be associated with substantial tissue damage characterised by oedema, demyelination and axonal loss [32, 33]. ‘T1-w hypointense lesions’ detected on volumetric spoiled gradient-recalled echo (SPGR), as used in the current and a growing number of other studies, have been less well characterised pathologically, but are likely to show similar correlations with underlying parenchymal damage.

A smaller number of rim lesions corresponded to ‘FLAIR-hypointense core lesions’, which have also been described in the literature [34], and are likely to represent a subset of ‘T1-w hypointense lesions’. Our data do not allow any firm conclusions to be drawn regarding the nature of these and their relationship to chronic low-grade inflammation.

Because of their apparent specificity for MS, the presence of rim lesions has been suggested as potentially useful in the radiological differential diagnosis of other neurological conditions whose imaging appearances may overlap with MS [15–20]. Although we have demonstrated their presence in early MS, the low prevalence would in practice limit diagnostic sensitivity.

In line with previous findings, we were unable to demonstrate strong evidence for a difference in the 1-year longitudinal volume evolution between rim lesions and non-rim lesions, [10, 13], although potential errors in our volumetric measurement due to manual segmentation of poorly defined individual lesions may limit the sensitivity of our analysis. We nevertheless observed that rim lesions were more commonly enlarging, in contrast to ‘shrinking’ non-rim lesions. Our finding of similar volume changes in non-rim lesions in subjects who had one or more rim lesions and those who had none may indicate a more localised inflammatory process in rim lesions.

A number of studies suggest that a subset of rim lesions may initially shrink [6, 10, 11]. While no clear relationship has been established between the presence of a paramagnetic rim and lesion growth measured over short intervals, recent findings suggest that differences in lesion volume evolution become more pronounced over longer time periods [10, 11]. Although it remains unclear whether rim lesions and slowly enlarging lesions define the same lesion subgroup, an overlap between the two is likely.

Our study focuses on patients with recently diagnosed RRMS, and the observation of overall shrinkage of WMLs may well reflect partial resolution of acutely swollen lesions over the year following the acute inflammatory episodes which originally lead to diagnosis. The early stage in disease course may also help to explain the lack of association observed between the presence of rim lesions and clinical disability, as substantial variation in disability between patients often only becomes apparent over several years. The lack of significant association detected between rim lesions and elevated plasma neurofilament levels may also be influenced by the modest prevalence of both in early disease, and the confound that the two measures were made a year apart.

Several factors can confound the assessment of paramagnetic phase rims, including high venous density, regions of juxtacortical signal heterogeneity and the presence of susceptibility or motion-related artefacts. While rims were more commonly observed across larger lesions, we were unable to assess hypointensity around the edge of smaller lesions reliably, which may in part be due to lower signal-to-noise ratio and contrast resolution at 3 T compared with ultra-high field 7 T. This challenge is compounded by the fact that lesions are sometimes only partially bordered by a rim of decreased signal on SWI. The difficulty in confidently classifying rim lesions is reflected in our limited inter-rater concordance in rim lesion count and accords with previous observations regarding the current lack of standardised methods for evaluating the presence of a paramagnetic phase rim, and that familiarity with susceptibility-based MRI may be necessary for guiding rim lesion assessment [30].

Our study has several limitations. First, the low prevalence of rim lesions in our patient cohort meant that relatively few lesions could be included in our analysis. A further significant limitation is that contrast-enhanced images were not acquired; as a result, ‘chronic active lesions’ that have been defined on imaging as non-gadolinium enhancing lesions with a paramagnetic rim could not be identified in our study. Active inflammatory lesions could also not be evaluated. We further recognise that SWI was only performed at a single 1-year timepoint (M12); the longitudinal evolution of the paramagnetic rim sign could therefore not be assessed and limits correlative measurements with neurofilament levels which were only sampled at baseline (M0). Future efforts will focus on collecting longitudinal SWI in the same cohort.

## Conclusion

Various lines of evidence indicate that rim lesions identified with SWI may reflect chronic inflammation in MS patients. We have demonstrated that rim lesions are also a feature of subjects with early MS using a 3 T SWI in a multi-centre context, and our data suggest a possible relationship with lesion growth and shrinkage. The integration of SWI rim lesions in routine MS imaging practice will require further studies in larger cohorts across the disease course and consensus on a standardised approach to their assessment.

## Supplementary Information

Below is the link to the electronic supplementary material.

Supplementary file1 (DOCX 1086 KB)

Supplementary file2 (DOCX 31 KB)

Supplementary file3 (DOCX 15 KB)

Supplementary file4 (DOCX 30 KB)

Supplementary file5 (DOCX 16 KB)

Supplementary file6 (DOCX 14 KB)

## Data Availability

Not applicable.

## Abbreviations

CIS: Clinically isolated syndrome
DICOM: Digital Imaging and Communications in Medicine
DMT: Disease-modifying therapy
EDSS: Expanded Disability Status Scale
FLAIR: Fluid-attenuated inversion recovery
FOV: Field of view
ICC: Intraclass correlation coefficient
ICV: Intracranial volume
MRI: Magnetic resonance imaging
MS: Multiple sclerosis
MSFC: Multiple Sclerosis Functional Composite
NIfTI: Neuroimaging Informatics Technology Initiative
NfL: Neurofilament
NMOSD: Neuromyelitis optica spectrum disorder
PASAT: Paced Auditory Serial Addition Test
RIS: Radiologically isolated syndrome
RRMS: Relapsing–remitting multiple sclerosis
SDMT: Symbol Digit Modalities Test
SPGR: Spoiled gradient-recalled echo
SWI: Susceptibility-weighted imaging;
T25FWT: Timed 25-Foot Walk Test
TE: Echo time
TI: Inversion time
TR: Repetition time
WML: White matter lesion
