# Supplementary material for: Rim lesions are demonstrated in early relapsing–remitting multiple sclerosis using 3 T-based susceptibility-weighted imaging in a multi-institutional setting
Source: Neuroradiology. 2021 Oct 19;64(1):109–17. doi: 10.1007/s00234-021-02768-x (PMC8724059; doi:10.1007/s00234-021-02768-x)
Supplement: Supplementary file 1 — Supplementary file1 (DOCX 1086 KB) [file 234_2021_2768_MOESM1_ESM.docx]

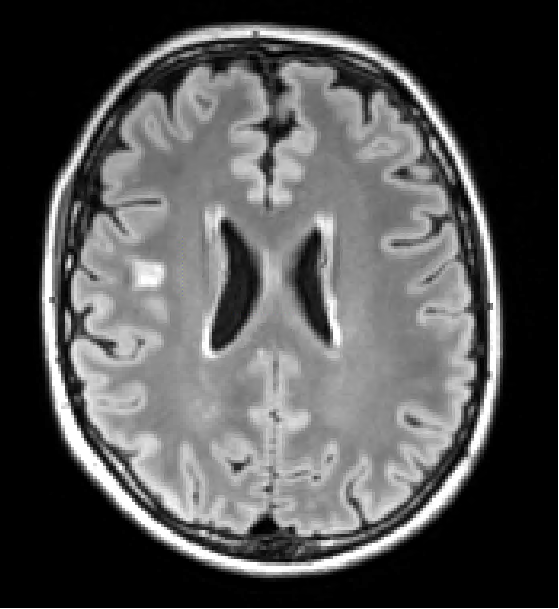

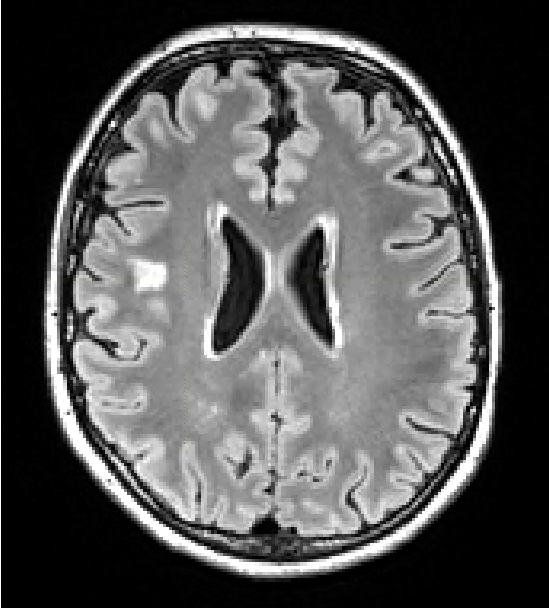


**a**

**b**

**Baseline**

**Follow-up**

**Supplementary Fig. S1** One-year longitudinal volume evolution of rim lesion. An increase in rim lesion size over the one-year period can be seen from side-by-side baseline (a) and follow-up (b) FLAIR images. Various terms have therefore been employed to describe these lesions, including ‘slowly enlarging lesions’, ‘slowly expanding lesions’ and ‘slowly evolving lesions’.
