# Supplementary material for: Rim lesions are demonstrated in early relapsing–remitting multiple sclerosis using 3 T-based susceptibility-weighted imaging in a multi-institutional setting
Source: Neuroradiology. 2021 Oct 19;64(1):109–17. doi: 10.1007/s00234-021-02768-x (PMC8724059; doi:10.1007/s00234-021-02768-x)
Supplement: Supplementary file 2 — Supplementary file2 (DOCX 31 KB) [file 234_2021_2768_MOESM2_ESM.docx]

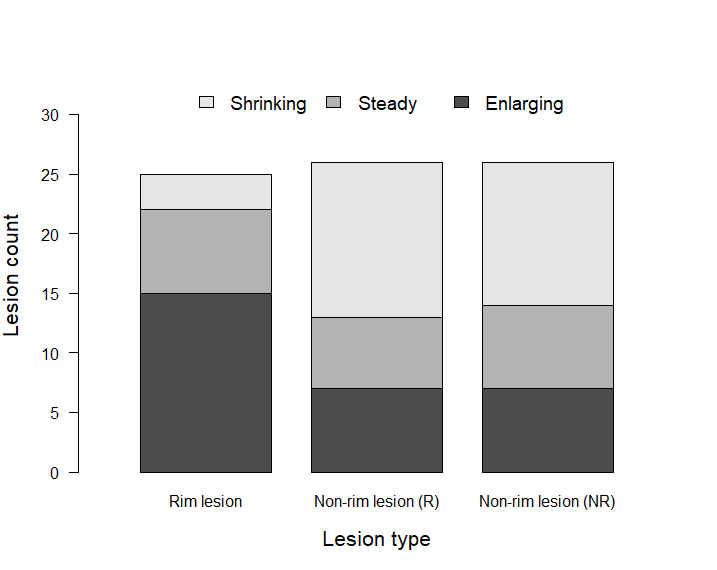


**Supplementary Fig. S2** Lesion categorisation. Barplots showing the number of shrinking, steady and enlarging lesions in rim lesions (3, 7 and 15 respectively), non-rim lesions in subjects with rim lesions (13, 6 and 7 respectively) and non-rim lesions in subjects without rim lesions (12, 7 and 7 respectively). Shrinking, steady and enlarging lesions are defined in the results section of the main manuscript.
