# Supplementary material for: Rim lesions are demonstrated in early relapsing–remitting multiple sclerosis using 3 T-based susceptibility-weighted imaging in a multi-institutional setting
Source: Neuroradiology. 2021 Oct 19;64(1):109–17. doi: 10.1007/s00234-021-02768-x (PMC8724059; doi:10.1007/s00234-021-02768-x)
Supplement: Supplementary file 3 — Supplementary file3 (DOCX 15 KB) [file 234_2021_2768_MOESM3_ESM.docx]

Supplementary Table S1. MRI acquisition parameters

| Main acquisition parameters for the 3T systems used in FutureMS | | | | |
| --- | --- | --- | --- | --- |
| Scan | T1 | T2 (dual echo) | 3D FLAIR | 2D FLAIR |
| Mode | 3D | 2D | 3D | 2D |
| FOV (mm) | 256 | 25.0 | 256 | 25.0 |
| Orientation | Sagittal vol | Axial | Sagittal vol | Axial |
| Slices | 1 slab (176) | 60 | 1 slab (176) | 60 |
| TR (ms) | 2500 | 3630 | 5000 | 9500 |
| TE (ms) | 2.26 | 9.6, 9.6 | 393 | 120 |
| TI (ms) | 1100 | n/a | 1800 | 2400 |
| Flip angle (degrees) | 7 | 150 | n/a | 150 |
| Phase encoding direction | A>>P | R>>L | A>>P | R>>L |
| Slice thickness (mm) | 1 | 3 | 1 | 3 |
| Acq matrix | 256 x 256 | 384 x 384 | 256 x 256 | 256 x 256 |
| Acq voxel size (mm) | 1 x 1 x 1 | 0.65 x 0.65 x 3 | 1 x 1 x 1 | 0.97 x 0.97 x 3 |
| Acq time (m:ss) | 5:59 | 3:58 | 6:52 | 4:47 |
