# Supplementary material for: Rim lesions are demonstrated in early relapsing–remitting multiple sclerosis using 3 T-based susceptibility-weighted imaging in a multi-institutional setting
Source: Neuroradiology. 2021 Oct 19;64(1):109–17. doi: 10.1007/s00234-021-02768-x (PMC8724059; doi:10.1007/s00234-021-02768-x)
Supplement: Supplementary file 4 — Supplementary file4 (DOCX 30 KB) [file 234_2021_2768_MOESM4_ESM.docx]

**Supplementary Table S2. Individual subject characteristics**

| Subject | Age (years) | Gender | Time from diagnosis to SWI (days) | Time from onset to SWI (days) | Duration of follow-up (days) | Follow-up EDSS | Baseline EDSS | Change in EDSS | T25FWT | PASAT | SDMT | MSFC | DMT | Rim lesion count | ‘T1-w hypointense lesion’ count | WML volume  (% ICV) | Baseline plasma NfL levels (pg/mL) |
| --- | --- | --- | --- | --- | --- | --- | --- | --- | --- | --- | --- | --- | --- | --- | --- | --- | --- |
| 1 | 47.0 | Female | 620 | 839 | 532 | 3 | 2 | 1 | 4.5 | 51 | 66 | 0.59 | Glatiramer acetate | 0 | 2 | 0.724 | 5.96 |
| 2 | 60.1 | Female | 569 | 1584 | 525 | 3.5 | 2 | 1.5 | 3.6 | 46 | 62 | 0.85 | Glatiramer acetate | 0 | 10 | 1.410 | 7.62 |
| 3 | 68.3 | Female | 480 | NA | 367 | 5.5 | 5 | 0.5 | 6.8 | 39 | 43 | -0.65 | NA | 0 | 30 | 1.792 | 12.27 |
| 4 | 37.2 | Female | 496 | 9669 | 412 | 2 | 2 | 0 | 3.8 | 50 | 62 | 0.59 | None | 0 | 19 | 1.524 | 5.07 |
| 5 | 57.4 | Male | 518 | 905 | 475 | 2 | 2 | 0 | 4.8 | 25 | 41 | -0.56 | Dimethyl fumarate | 0 | 14 | 1.500 | 10.35 |
| 6 | 42.5 | Male | 517 | 2483 | 468 | 6 | 6 | 0 | 8.1 | 15 | 26 | -1.76 | None | 0 | 39 | 2.252 | 10.21 |
| 7 | 46.5 | Female | 472 | 6471 | 442 | 3 | 4 | -1 | 3.8 | 59 | 67 | 0.88 | None | 0 | 32 | 2.160 | 13.95 |
| 8 | 39.4 | Female | 577 | 1168 | 421 | 3.5 | 2.5 | 1 | 5 | 54 | 43 | 0.10 | Alemtuzumab | 0 | 7 | 0.201 | 3.21 |
| 9 | 50.9 | Female | 534 | 3083 | 507 | 4 | 3.5 | 0.5 | 3.6 | 37 | 58 | 0.30 | Azathioprine | 0 | 4 | 0.922 | 6.70 |
| 10 | 26.9 | Male | 538 | 970 | 519 | 2 | 0 | 2 | 4 | 0 | 67 | -0.57 | Dimethyl fumarate | 0 | 8 | 0.619 | 2.64 |
| 11 | 57.9 | Female | 515 | 2310 | 428 | 2.5 | 2.5 | 0 | 4.3 | 56 | 59 | 0.69 | None | 1 | 10 | 0.345 | 4.31 |
| 12 | 52.6 | Female | 487 | 1927 | 392 | 6 | 2 | 4 | 7.6 | 43 | 53 | -0.83 | Dimethyl fumarate | 2 | 15 | 1.019 | NA |
| 13 | 27.5 | Female | 483 | NA | 414 | 1 | 1 | 0 | 3.7 | 38 | 58 | 0.38 | Interferon beta‑1a | 0 | 9 | 0.498 | 16.47 |
| 14 | 34.9 | Female | 583 | 887 | 512 | 3 | 2 | 1 | 4.2 | 33 | 47 | 0.06 | None | 0 | 3 | 0.137 | 12.10 |
| 15 | 26.0 | Male | 529 | 925 | 400 | 3.5 | 3 | 0.5 | 4.6 | 41 | 74 | -0.02 | Other | 1 | 15 | 0.734 | 10.43 |
| 16 | 35.2 | Female | 436 | 630 | 408 | 3 | 3.5 | -0.5 | 5.3 | 45 | 64 | -0.05 | Glatiramer acetate | 0 | 19 | 0.311 | 6.90 |
| 17 | 31.9 | Female | 606 | 910 | 415 | 2 | 1.5 | 0.5 | 4.7 | 40 | 69 | 0.21 | Dimethyl fumarate | 0 | 5 | 0.839 | 2.86 |
| 18 | 57.0 | Female | 648 | NA | 455 | 3.5 | 3 | 0.5 | 4.6 | 57 | 53 | 0.34 | None | 0 | 31 | 0.644 | 5.27 |
| 19 | 28.5 | Female | NA | NA | 451 | 1 | 2 | -1 | 3.5 | 59 | 66 | 1.17 | Alemtuzumab | 0 | 6 | 0.159 | 7.56 |
| 20 | 50.3 | Female | 484 | 722 | 450 | 6 | 5.5 | 0.5 | 12.1 | 30 | 28 | -1.62 | NA | 8 | 116 | 3.212 | 12.37 |
| 21 | 55.2 | Male | 415 | NA | 381 | 1.5 | 2 | -0.5 | 4.2 | 52 | 52 | 0.20 | None | 0 | 22 | 1.242 | 18.05 |
| 22 | 51.3 | Female | 478 | 1351 | 402 | 2 | 1.5 | 0.5 | 4.3 | 48 | 69 | 1.10 | Other | 0 | 15 | 0.821 | 4.91 |
| 23 | 23.2 | Male | 446 | 947 | 386 | 2.5 | 1 | 1.5 | 3.9 | 60 | 75 | 0.72 | Dimethyl fumarate | 1 | 31 | 1.044 | 9.77 |
| 24 | 45.6 | Female | 451 | 2606 | 385 | 3 | 2.5 | 0.5 | 5.5 | 55 | 56 | 0.39 | Glatiramer acetate and dimethyl fumarate | 0 | 16 | 0.608 | 4.95 |
| 25 | 52.7 | Female | 414 | 2676 | 368 | 4.5 | 3.5 | 1 | 5.3 | 49 | 40 | 0.12 | Dimethyl fumarate | 2 | 32 | 1.528 | 5.96 |
| 26 | 51.0 | Female | 422 | NA | 365 | 3 | 3 | 0 | 6.1 | 48 | 45 | -0.32 | Interferon beta‑1a | 0 | 42 | 1.214 | 2.66 |
| 27 | 38.8 | Male | 504 | NA | 470 | 1 | 2 | -1 | 4.1 | 58 | 50 | 0.83 | Other | 1 | 14 | 0.681 | 4.19 |
| 28 | 65.9 | Female | 498 | NA | 404 | 6 | 6 | 0 | 7.3 | 30 | 43 | -0.88 | None | 0 | 27 | 2.269 | 9.34 |
| 29 | 31.3 | Male | 459 | 1656 | 367 | 2 | 2 | 0 | 4.4 | 44 | 67 | 0.12 | None | 1 | 14 | 0.776 | 4.17 |
| 30 | 26.3 | Female | 525 | 705 | 427 | 2.5 | 2 | 0.5 | 5.2 | 41 | 63 | 0.27 | Dimethyl fumarate | 3 | 25 | 1.346 | 16.55 |
| 31 | 53.4 | Female | 611 | 1457 | 371 | 2.5 | 2 | 0.5 | 4 | 51 | 57 | 0.76 | None | 0 | 13 | 0.967 | 5.50 |
| 32 | 49.1 | Female | 391 | 966 | 369 | 2 | 1.5 | 0.5 | 3.9 | 49 | 63 | 1.14 | Glatiramer acetate | 0 | 8 | 0.758 | 6.01 |
| 33 | 35.9 | Female | 470 | NA | 430 | 4.5 | 3 | 1.5 | 6.3 | 23 | 51 | -0.83 | Dimethyl fumarate | 0 | 22 | 1.437 | 11.40 |
| 34 | 43.1 | Female | 400 | 2353 | 392 | 2.5 | 3 | -0.5 | 4.1 | 40 | 51 | -0.18 | Dimethyl fumarate and azathioprine | 0 | 5 | 0.248 | 4.80 |
| 35 | 22.0 | Male | 413 | 777 | 366 | 1.5 | 3 | -1.5 | 5.7 | 26 | 48 | -0.13 | Other | 0 | 15 | 1.179 | 24.06 |
| 36 | 25.1 | Female | 560 | 2066 | 363 | 3.5 | 3.5 | 0 | 4.2 | 53 | 72 | 0.73 | None | 0 | 2 | 0.120 | 23.18 |
| 37 | 41.9 | Female | 541 | NA | 407 | 3.5 | 3 | 0.5 | 4.6 | 54 | 38 | 0.44 | Dimethyl fumarate | 1 | 38 | 1.301 | 4.51 |
| 38 | 25.6 | Female | 513 | 1121 | 382 | 3 | 2 | 1 | 4.6 | 45 | 67 | 0.68 | Dimethyl fumarate | 0 | 13 | 0.157 | 5.65 |
| 39 | 50.5 | Female | 445 | NA | 385 | 2.5 | 2.5 | 0 | 4.7 | 29 | 53 | 0.03 | Interferon beta‑1a | 0 | 5 | 0.702 | 5.03 |
| 40 | 31.0 | Male | 392 | 796 | 371 | 3 | 2.5 | 0.5 | 4.3 | 58 | 62 | 0.73 | Dimethyl fumarate | 1 | 19 | 0.619 | 10.67 |
| 41 | 49.3 | Female | 441 | 588 | 365 | 1.5 | 2.5 | -1 | 3.8 | 59 | 64 | 1.33 | None | 0 | 39 | 1.154 | 7.71 |
| 42 | 44.2 | Female | 491 | 606 | 366 | 2 | 2 | 0 | 4.5 | 54 | 62 | 0.82 | None | 0 | 4 | 0.132 | 4.09 |
| 43 | 60.2 | Female | NA | NA | 366 | 3 | 2 | 1 | 4.5 | 44 | 55 | 0.42 | None | 0 | 47 | 1.259 | 7.80 |
| 44 | 23.3 | Male | 382 | 840 | 368 | 1 | 1.5 | -0.5 | 5.3 | 56 | 67 | 0.51 | Dimethyl fumarate | 3 | 12 | 0.281 | 4.55 |

NA – Not available
