# Supplementary material for: Rim lesions are demonstrated in early relapsing–remitting multiple sclerosis using 3 T-based susceptibility-weighted imaging in a multi-institutional setting
Source: Neuroradiology. 2021 Oct 19;64(1):109–17. doi: 10.1007/s00234-021-02768-x (PMC8724059; doi:10.1007/s00234-021-02768-x)
Supplement: Supplementary file 5 — Supplementary file5 (DOCX 16 KB) [file 234_2021_2768_MOESM5_ESM.docx]

Supplementary Table S3. Lesion selection

| Subject | Rim lesions | Non-rim lesions (R) | Non-rim lesions (NR) |
| --- | --- | --- | --- |
| 1 | 0 | 0 | 0 |
| 2 | 0 | 0 | 1 |
| 3 | 0 | 0 | 2 |
| 4 | 0 | 0 | 1 |
| 5 | 0 | 0 | 2 |
| 6 | 0 | 0 | 2 |
| 7 | 0 | 0 | 2 |
| 8 | 0 | 0 | 0 |
| 9 | 0 | 0 | 0 |
| 10 | 0 | 0 | 0 |
| 11 | 1 | 1 | 0 |
| 12 | 2 | 2 | 0 |
| 13 | 0 | 0 | 1 |
| 14 | 0 | 0 | 0 |
| 15 | 1 | 2 | 0 |
| 16 | 0 | 0 | 1 |
| 17 | 0 | 0 | 1 |
| 18 | 0 | 0 | 0 |
| 19 | 0 | 0 | 1 |
| 20 | 8 | 8 | 0 |
| 21 | 0 | 0 | 1 |
| 22 | 0 | 0 | 1 |
| 23 | 1 | 2 | 0 |
| 24 | 0 | 0 | 1 |
| 25 | 2 | 2 | 0 |
| 26 | 0 | 0 | 1 |
| 27 | 1 | 0 | 0 |
| 28 | 0 | 0 | 1 |
| 29 | 1 | 1 | 0 |
| 30 | 3 | 3 | 0 |
| 31 | 0 | 0 | 1 |
| 32 | 0 | 0 | 0 |
| 33 | 0 | 0 | 1 |
| 34 | 0 | 0 | 0 |
| 35 | 0 | 0 | 1 |
| 36 | 0 | 0 | 0 |
| 37 | 1 | 1 | 0 |
| 38 | 0 | 0 | 0 |
| 39 | 0 | 0 | 0 |
| 40 | 1 | 1 | 0 |
| 41 | 0 | 0 | 2 |
| 42 | 0 | 0 | 0 |
| 43 | 0 | 0 | 2 |
| 44 | 3 | 3 | 0 |
| Total | 25 | 26 | 26 |
