# Supplementary material for: Rim lesions are demonstrated in early relapsing–remitting multiple sclerosis using 3 T-based susceptibility-weighted imaging in a multi-institutional setting
Source: Neuroradiology. 2021 Oct 19;64(1):109–17. doi: 10.1007/s00234-021-02768-x (PMC8724059; doi:10.1007/s00234-021-02768-x)
Supplement: Supplementary file 6 — Supplementary file6 (DOCX 14 KB) [file 234_2021_2768_MOESM6_ESM.docx]

Supplementary Table S4. MRI hardware for 3T systems

|  | Edinburgh | Glasgow | Dundee |
| --- | --- | --- | --- |
| Scanner manufacturer | Siemens | Siemens | Siemens |
| Scanner model | Prisma | Prisma | Prisma |
| Field | 3.0T | 3.0T | 3.0T |
| Head coil | 32 ch | 20 ch | 20 ch |
| Max gradients | 80 mT/m | 80 mT/m | 80 mT/m |
